# T Regulatory Cells in the Visceral Adipose Tissues

**DOI:** 10.20900/immunometab20220002

**Published:** 2021-12-22

**Authors:** Allen N. Fooks, Louise M. D’Cruz

**Affiliations:** Department of Immunology, University of Pittsburgh, Pittsburgh, PA 15213, USA

**Keywords:** Tregs, adipose tissue, metabolism, obesity

## Abstract

CD4^+^ Foxp3^+^ T regulatory cells (Tregs) residing in the visceral adipose tissues (VAT) have profound effects on local and systemic metabolism. Although many of the molecular characteristics of VAT resident Tregs have been identified, *how* these cells promote metabolic homeostasis is still unclear. Several new publications help to illuminate the molecular mechanisms that underpin VAT resident Treg function and will be discussed here.

Recent published data have clearly shown that CD4^+^ Foxp3^+^ regulatory T cells (Tregs) possess distinct tissue specificity, resulting in Tregs with unique tissue-specific functions depending on their tissue of residence [1-7]. In the adipose tissue depots, particularly in the visceral adipose tissue (VAT), Tregs have been identified as transcriptionally distinct and clonally restricted and become depleted in obese mice and humans [4,8-13]. Short-term ablation of Tregs resulted in increased adipose tissue inflammatory gene expression, insulin resistance and glucose intolerance in high fat diet (HFD)-fed mice [4,14], indicating that Tregs are beneficial in repressing inflammation in adipose tissue. Thus, VAT resident Tregs may hold the key to the design of new therapeutics for the treatment of obesity, which affects >40% of the adult US population [15,16].

A recent publication by Beppu et al. shed light on how VAT resident Tregs function to control adipose tissue inflammation [17]. In this study, the cytokine Interleukin-10 (IL-10) or its upstream transcriptional regulator Blimp-1 were conditionally ablated in Tregs. Rather than increasing weight gain and insulin resistance, these conditional knockout mice were protected from high fat diet weight gain. Mechanistically, IL-10 was reported to suppress adipocyte thermogenesis [18,19], the process in which white adipocytes can convert stored chemical energy into heat, in response to stimulation such as cold or exercise [20,21]. Thus, ablation of IL-10 secretion by Tregs resulted in increased adipocyte thermogenesis leading to reduced weight gain and improvements in insulin resistance in the Treg-specific conditionally deficient mice [17] (Figure 1). These data showed that the function of VAT resident Tregs is more complex and multifaceted than previously considered and raised the interesting notion that it is communication between Tregs and non-hematopoietic cells that help to shape VAT and metabolic homeostasis.

A new publication also investigated how adipose tissue resident Tregs communicate with adipocytes to affect metabolism and body weight homeostasis [22]. Li and colleagues published that Tregs recently migrated from the secondary lymphoid tissues to the VAT and subcutaneous adipose tissue (SAT) express the protein CD73, a surface enzyme that converts AMP to adenosine [22]. Adenosine is used by proximal adipocytes to increase adipocyte thermogenesis [23,24]. These recently emigrated Tregs also express low levels of ST2, the high affinity Interleukin-33 (IL-33) receptor [22]. This study revealed that CD73^+^ ST2^−^ Tregs differentiate into mature CD73^−^ ST2^+^ adipose tissue resident Tregs through an insulin signaling-driven Hif1α-Med23-PPARγ pathway [22]. Indeed, deletion of the insulin receptor on Tregs substantially reduced PPARγ expression in VAT and SAT resident Tregs (PPARγ expression is a hallmark of VAT resident Tregs [9]) and prevented differentiation of the cells to CD73^−^ ST2^+^ Tregs [22]. Similarly, conditional ablation of either Hif1α or Med23 in Tregs also prevented differentiation of adipose tissue resident Tregs to CD73^−^ ST2^+^ Tregs [22]. While insulin signaling was shown to be directly responsible for converting CD73^+^ ST2^−^ Tregs to terminally differentiated CD73^−^ ST2^+^ Tregs, IL-33 was required to maintain Treg homeostasis and the expansion of the CD73^−^ ST2^+^ population [22,25]. Finally, this study showed that increased adenosine production in insulin receptor, Hif1α or Med23 Treg-specific deficient mice increased thermogenesis gene expression and improved glucose tolerance, owing to the increased CD73^+^ ST2^−^ Tregs within the adipose tissues [22] (Figure 1). One potential caveat with these new data is that the CD73^+^ ST2^−^ and CD73^−^ ST2^+^ Tregs populations were identified using pooled VAT and SAT. It is possible that the visceral and subcutaneous adipose tissue depots have distinct Treg subsets residing each, and this will no doubt be clarified in future studies. Moreover, determining if Blimp-1 and IL-10 expression are primarily restricted to the CD73^−^ ST2^+^ adipose tissue Tregs will help to improve our understanding of the complex life cycle and function of adipose tissue resident Tregs.

One curious aspect of VAT resident Tregs is that their frequency is reduced after diet induced obesity (DIO) and during aging [4,22,26,27]. Addition of Tregs to older mice via adoptive transfer was shown to be detrimental, indicating that with VAT resident Tregs, timing and context are critical and that reduction in the frequency of VAT resident Tregs may be beneficial during aging [26]. One possible mechanism that has been recently suggested for why VAT resident Tregs are reduced during high fat diet feeding is an increase in the soluble isoform of ST2, sST2, which functions as a decoy receptor [28]. High expression of sST2 prevented IL-33-ST2 signaling in VAT resident Tregs, a process that is critical for their survival [25,29]. Indeed, Li and colleagues showed that CD73^−^ ST2^+^ Tregs decline with long-term DIO and age, and that this decline correlates with an increase in sST2 expression [22].

Finally, Li and colleagues focused instead on Interferon-α (IFN-α) and its role in the depletion of VAT resident Tregs in long term DIO in mice [27]. The authors carefully examined the frequency and number of VAT resident Tregs, using the vTreg53-tg mouse model, previously developed by the authors, in standard and high fat diet fed mice over time [27]. In mice on normal chow, the frequency and number of VAT resident Tregs consistently increased over a 16-week time-period [27]. In contrast, feeding mice a high fat diet from 12 weeks of age led to a significant increase in VAT Tregs after 4 weeks, followed by rapid loss of the VAT Treg population between weeks 8 and 16 [27]. This study further investigated the mechanisms underlying the rapid loss of VAT Tregs during DIO. Differential gene expression analysis of VAT Tregs in DIO mice revealed upregulation of genes associated with responses to pro-inflammatory cytokines and downregulation of genes required for lipid metabolism [27]. This study showed that VAT resident Tregs are highly sensitive to IFN-α and that increasing concentrations of IFN-α perturbed the turnover and survival of VAT resident Tregs [27] (Figure 1). Finally, it was revealed that plasmacytoid dendritic cells (pDCs) are the primary source of IFN-α in the VAT and that the frequency of pDCs that secrete IFN-α is increased with continuous long-term high fat diet feeding.

In conclusion, these three recent studies have helped to illuminate how Tregs function in the adipose tissues in mice. VAT Treg specific IL-10 secretion represses adipocyte thermogenesis while adenosine helps to increase thermogenesis [17,22]. We also have a greater understanding of the mechanisms by which VAT resident Tregs become depleted during high fat diet feeding. As DIO in mice and humans results in decreased frequency of anti-inflammatory macrophages and conversely a substantial increase in inflammatory macrophages in the adipose tissue depots, further study will be required to investigate if loss of CD73^−^ ST2^+^ VAT Tregs in HFD fed mice is correlative or causative in inflammatory macrophage polarization. Finally, although these newest insights into VAT Treg biology have been obtained with murine models, they present a roadmap to test the function of VAT Tregs in humans and offer the potential for eventual design of therapies to treat DIO and obesity in people.

## Figures and Tables

**Figure 1.**
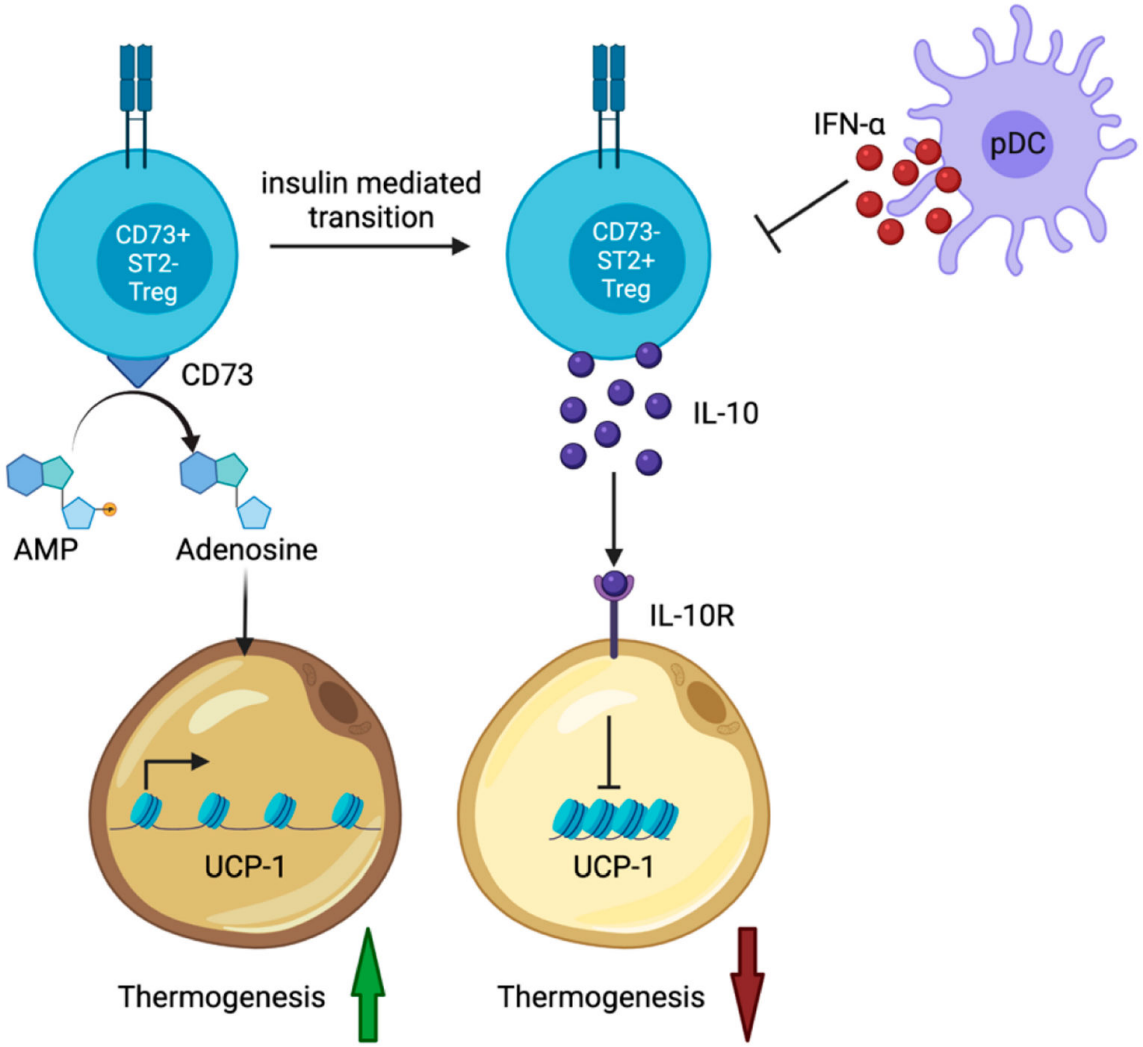
Schematic of VAT resident Treg differentiation, survival, and regulation of adipocyte thermogenesis. Newly published data have shown that Tregs enter VAT as CD73^+^ ST2^−^ cells and that insulin signaling initiates differentiation of these cells to CD73^−^ ST2^+^ VAT resident Tregs. Blimp-1 driven IL-10 secretion by ST2^+^ VAT Tregs was shown to repress adipocyte thermogenesis while adenosine conversion by CD73^+^ VAT Tregs was shown to promote adipocyte thermogenesis. Under high fat diet conditions leading to diet induced obesity (DIO), it was revealed that ST2^+^ VAT Tregs are susceptible to IFN-α secretion by plasmacytoid dendritic cells (pDCs) which promotes VAT Treg death and represses proliferation.
